# Understanding potentially avoidable emergency department visits in older adults: a survey with healthcare professionals across in- and outpatient care in Sweden

**DOI:** 10.1136/bmjopen-2026-121668

**Published:** 2026-08-14

**Authors:** Hanna Bring, Monica Bergqvist, Lars L Gustafsson, Anikó Veg, Katarina Bohm, Karin Modig, Pia Bastholm-Rahmner, Katharina Schmidt-Mende

**Affiliations:** 1 Division of Family Medicine and Primary Care, Department of Neurobiology, Care Sciences and Society, Karolinska Institutet, Huddinge, Sweden; 2 Academic Primary Care Centre, Stockholm, Sweden; 3 Division of Nursing, Department of Neurobiology, Care Sciences and Society, Karolinska Institutet, Huddinge, Sweden; 4 Karolinska University Hospital, Division of Clinical Pharmacology, Department of Laboratory Medicine, Karolinska Institutet, Stockholm, Sweden; 5 Independent Author, Stockholm, Sweden; 6 Department of Health Sciences, Swedish Red Cross University, Huddinge, Sweden; 7 Department of Clinical Science and Education, Södersjukhuset, Karolinska Institute, Stockholm, Sweden; 8 Unit of Epidemiology, Institute of Environmental Medicine, Karolinska Institutet, Stockholm, Sweden

**Keywords:** Emergency Service, Hospital, Primary Health Care, Aged, Multimorbidity, Surveys and Questionnaires

## Abstract

**Abstract:**

**Objectives:**

Older adults with multiple long-term conditions account for a large share of emergency department (ED) visits and up to 25% may be avoidable. Efforts to reduce such visits show mixed results, likely due to multiple caregivers and limited adaptation to local contexts. We explored factors contributing to avoidable ED visits as reported by multiple care providers, focusing on modifiable factors of importance in the design of a sustainable primary care (PC) intervention.

**Design and setting:**

Web-based survey conducted across six in- and outpatient healthcare providers in Stockholm, Sweden: ED, geriatrics, ambulance services, advanced home-based healthcare, PC and home healthcare (both within and outside regular working hours). Closed-ended questions were analysed descriptively and open-ended questions using qualitative content analysis.

**Participants:**

A total of 100 healthcare professionals (nurse assistants, registered nurses and physicians) with at least 6 months of working experience.

**Results:**

All providers considered potentially avoidable ED visits a major concern, with home care workers seen as the main initiators of ED referrals. One overarching theme was generated—medical and psychosocial complexities in the assessment of older patients with multiple long-term conditions—and four categories of contributing factors: (1) insufficient access to health and social care, (2) poor communication and cooperation, (3) deficient professional competence and geriatric knowledge and (4) unclear responsibility and fragmented care. Five providers highlighted limited access to care as the most demanding concern while PC noted poor communication.

**Conclusions:**

An intervention should combine enhanced home-based care, better collaboration across providers, direct geriatric admissions and targeted training for home care workers. Accounting for differences in provider perspectives in intervention design may enhance feasibility and acceptability.

**STRENGTHS AND LIMITATIONS OF THIS STUDY**

- Inclusion of healthcare professionals across six in- and outpatient providers and multiple levels of care enabled a comprehensive, system-wide perspective on factors contributing to potentially avoidable emergency department visits.
- The sample included experienced respondents (≈70% with >10 years of experience) and multiple professional groups (physicians, nurses, nurse assistants), strengthening the relevance and breadth of perspectives.
- The use of a web-based survey with open-ended questions allowed respondents to express their views freely. However, variation in response length limited depth and interpretability for short responses.
- Quota sampling of healthcare providers supported diversity of perspectives but does not ensure representativeness.
- Small numbers of respondents in some groups limit generalisability; quantitative findings should therefore be interpreted as hypothesis-generating.

## Background

 Older adults account for up to 40% of all emergency department (ED) visits globally,^1–4^ and in Sweden, approximately half of adults aged 65 years or older sought ED care in 2023.^5^ While vital in emergencies, ED visits pose risks for frail older adults and are associated with complications such as infections, medication errors, functional decline and mortality.^6–8^ The anticipated rise in ED visits due to an ageing population^9 10^ is a global public health concern, causing strain to hospitals and EDs.^1–4 11 12^

Every fourth ED visit in older adults might be avoidable, either with care at another level or through more proactive outpatient care.^13^ Since ED visits expose older adults to unnecessary risks,^3 6 7^ reducing them has been a focus of previous research.^14–16^ Yet, the interventions show inconsistent results.^14–16^ Interventions based in primary care (PC) appear more promising than those based in hospitals,^14 15^ particularly when including comprehensive geriatric assessment, home visits and care coordination.^14 16^ However, the overall evidence remains inconclusive,^14 16 17^ and it is unclear to what extent these interventions have been implemented and are sustainable in clinical practice. We are not aware of any recent randomised controlled trials conducted in Scandinavia.

The inconsistent results may reflect the presence of multiple potentially effective components within complex interventions, as well as insufficient adaptation to local contexts.^14^ Additionally, the care chain often involves multiple caregivers, yet previous research has rarely incorporated perspectives from all stakeholders, resulting in an incomplete understanding of the factors behind potentially avoidable ED visits.^18–20^

Overall, more interventional research targeting frail older adults is crucial to prepare healthcare systems for an ageing population^16^ in general, and to reduce the adverse outcomes associated with ED visits in particular. Understanding which intervention components are effective and how context influences them is crucial, as highlighted in the Medical Research Council framework for complex healthcare interventions.^21^ Therefore, this study explores factors contributing to potentially avoidable ED visits among older adults with multiple long-term conditions, focusing on modifiable factors and incorporating perspectives from multiple care providers to ensure a comprehensive understanding. The findings will help to inform the design of a sustainable PC-based intervention to reduce such visits.

## Methods

### Study design

A web-based survey with closed- and open-ended questions was conducted to capture diverse perspectives from healthcare professionals across care settings on potentially avoidable ED visits among older adults with multiple long-term conditions. The open-ended questions allowed respondents to elaborate in their own words, facilitating a broad exploration of the phenomenon.^22^

### Study setting

The study was conducted in urban and suburban areas of Stockholm (population 2.5 million). In Sweden, health and social care is tax funded. Regions organise healthcare, while municipalities organise social care. PC does not function as a gatekeeper for ED visits, and patients can access EDs without referral. Municipalities employ home care workers who provide support with instrumental and personal activities of daily living. They may be formally trained nurse assistants or untrained personnel.

The survey included healthcare professionals from six key healthcare providers:

1. ED: 24/7 emergency care with full diagnostic capacity; no referral required.
2. Geriatrics: Hospital-based geriatric wards for acute care, with limited access to advanced diagnostics, particularly out of hours.
3. Ambulance services: Community emergency assessments 24/7, mainly staffed by registered nurses.
4. Advanced home-based healthcare: 24/7 services providing hospital-level care at home, including intravenous therapy and blood transfusions, with on-call physicians.
5. PC: Clinic-based care and basic home healthcare delivered by teams of GPs, registered nurses, and nurse assistants. Home healthcare includes wound care, medication administration, and medical assessment and follow-up for homebound patients.
6. Home healthcare out of hours (HHC): Evening, night, and weekend services providing basic nursing care (eg, wound care, medication administration), staffed by registered nurses and nurse assistants without on-call physicians.

### Survey development

The survey was developed by a panel of physicians, registered nurses, a pharmacist, a pedagogue and a behavioural scientist. All panel members had expertise in healthcare development processes, except for HB (MD, PhD student). The panel contributed to development through iterative discussions on content, clarity and relevance from both clinical and methodological perspectives. To enhance face validity, the survey was pretested on three professionals (from ED, geriatrics and advanced home-based healthcare), resulting in minor adjustments of style and grammar.

The survey included three open-ended questions with free-text answers, three closed-ended questions with predefined options (multiple-choice, rating scales) and demographic information (online supplemental material 1). The cover page provided a definition of the target population to ensure common understanding:

‘Older adults with multiple long-term conditions’ refers to older individuals with complex medical and care needs.

The three closed-ended questions were:

1. How significant is the problem of older adults with multiple long-term conditions visiting the ED when such visits could have been prevented?
2. Who is most likely to initiate an ED visit for an older person with multiple long-term conditions?
3. How does your collaboration with other care providers work regarding older adults with multiple long-term conditions?

The three open-ended questions were:

1. Describe a patient case with an older adult with multiple long-term conditions where it would have been possible to prevent an ED visit. What should have been done differently and how?
2. What changes need to be made in healthcare to ensure that older adults with multiple long-term conditions avoid unnecessary ED visits?
3. Describe three or more things that you find difficult or challenging in working with older adults with multiple long-term conditions.

### Recruitment and data collection

Quota sampling was used to ensure representation from all six healthcare providers.^23^ In qualitative studies, quota sampling is particularly useful for capturing specific perspectives and experiences and allows for a more targeted selection of participants while still maintaining diversity within the sample.^23^

Previous research indicates that 20 respondents are sufficient to encompass the potential variation in how a phenomenon is experienced in qualitative research.^24^ Using the concept of information power,^25^ we determined our aim to be narrow and that dense sample specificity could be achieved by recruiting experienced respondents, supporting a lower sample size. Therefore, we considered 20 respondents from each provider to be adequate to address our research objectives.

The inclusion criterion of respondents was at least 6 months of working experience. The main professional groups targeted were physicians, registered nurses and nurse assistants. They are all key healthcare providers involved in the care of older adults with multiple long-term conditions and in decisions on care related to ED visits. Respondents were recruited through managers and key contacts within each provider group. The managers and key contacts were identified via authors’ professional networks. Key contacts were informed about the study and asked to invite 20 eligible healthcare professionals with experience of the issue and interest in elaborating their responses to complete the survey. Staff were invited to participate via email. No incentive was offered.

The survey was initially sent to the respondents between September 2022 and January 2023. A cover letter with information about the study, its confidentiality, the voluntary nature of participation and informed consent was included at the beginning of the survey (online supplemental material 1). Although 20 respondents per target group initially agreed to participate, response rates varied. After five reminder e-mails to eligible respondents, additional respondents were recruited through the same key contacts, who distributed the survey link directly.

Initially, PC professionals were excluded, as their perspectives were to be studied separately, but following preliminary analysis this group was included to gain a more comprehensive understanding. As PC practices are typically smaller organisations, a different recruitment strategy was used. The survey link was disseminated widely to PC professionals via key contacts and authors’ professional networks between November 2023 and February 2024.

Due to recruitment difficulties and time restrictions, fewer than 20 respondents were included in four of six healthcare providers. The aim was to recruit approximately 20 respondents from each healthcare provider. As the survey link was disseminated through key contacts to facilitate recruitment, the total number of eligible recipients could not be established, and a response rate could therefore not be calculated. However, in line with the qualitative design, the focus was on obtaining sufficiently rich and diverse data rather than achieving a specific response rate. Using the concept of information power,^24^ we assessed the data as sufficiently rich in information in relation to the study’s aim, providing adequate nuance and diversity for analysis.

### Patient and public involvement statement

The research idea emerged during interview studies with frail older adults (another manuscript submitted).^26^ Healthcare professionals pretested the survey. The findings will inform the development of a PC intervention, for which patients and the public are actively involved in the co-design process.

### Data analysis

#### Analyses of closed-end questions

Descriptive statistics (means, frequency, percentages) were used to describe the respondents’ demographics and the closed-ended questions with predefined answers from the survey. Proportions for multiple-choice questions were calculated using the total number of responses as the denominator.

#### Analyses of open-ended questions

The open-ended questions were analysed using qualitative content analysis,^27^ with an inductive approach.^28^ As open-ended questions can generate diverse responses, content analysis was chosen to enable flexible data reduction and categorisation.^27^ The analysis was conducted manually.

Primary qualitative analysis was conducted by PB-R (senior researcher with expertise in qualitative research, behavioural scientist) and MB (senior researcher with expertise in qualitative research, registered nurse). After recruitment of PC respondents, HB (PhD student, MD, resident in general practice) took main responsibility for splitting responses into meaning units, coding and analysis, with regular meetings and support from MB and PB-R.

The free-text responses from the six providers were first analysed separately and then combined. First, the free-text responses were read repeatedly to gain an overall understanding. Second, we identified meaning units^27^ in respondents’ responses relevant to the study aim. Responses containing multiple aspects were split into separate meaning units, which were condensed and coded while preserving their interpreted meaning. Codes were grouped into categories of factors contributing to potentially avoidable ED visits (online supplemental material 2). Finally, an overarching theme was derived from the data and interpreted as an underlying context. Participants were not invited to provide feedback on findings.

To explore differences in perspectives across providers, the number of meaning units per category and provider was counted.^27^ Because longer responses were divided into several meaning units, a single respondent could contribute multiple meaning units to the same category. The proportions of meaning units assigned to each category were then calculated for each provider.

To illustrate the complexity of the results and how the contributing factors are interconnected, a patient case as narrated by one of the respondents is presented at the end of the results section. The authors expanded shorthand (eg, ‘pat’ to ‘patient’) into full sentences without altering the original content.

### Rigour

We followed the Consolidated criteria for Reporting Qualitative research (COREQ) checklist for reporting when applicable.^29^ The qualitative data analysis was conducted by three researchers (HB, MB, PB-R). Authors identified patterns through reciprocal reading of responses and categories, which is a way to increase trustworthiness in the analytic process.^27 30^ Throughout the analysis process, the researchers engaged in continuous reflexive discussions, critically examining how their professional backgrounds and preconceptions could influence data interpretation. Reflexive team discussions were used to enhance awareness of these influences and to challenge and refine emerging interpretations. In addition, preliminary findings were reviewed and discussed within the research team (KB, professor and registered nurse; AV, senior researcher and pedagogue) to ensure that interpretations were not overlooking relevant content and to enhance analytical credibility.

## Results

The results are presented in four parts: quantitative findings from closed-ended questions, qualitative analysis of open-ended questions, differences in provider perspectives and a patient case narrated by a respondent.

### Quantitative findings from closed-ended questions

A total of 100 healthcare professionals from six different healthcare providers completed the survey (table 1). Respondents were predominantly female (67%) and registered nurses (67%), with an age range from 25 to 63 years and varied work experience.

**Table 1:** Demographic and professional characteristics of participants across care settings

Characteristic	Total (n=100)	Emergency department (n=23)	Geriatrics (n=10)	Ambulance services (n=15)	Advanced home-based healthcare (n=18)	Primary care and home healthcare (n=22)	Home healthcare out-of-hours (n=12)
Gender, n (%)							
Female	67 (67)	13 (57)	9 (90)	5 (33)	13 (72)	18 (82)	9 (75)
Other/no answer	4 (4)	1 (4)	0 (0)	0 (0)	1 (6)	0 (0)	2 (17)
Age, years							
Mean (min–max)	44 (25–63)	41 (25–63)	43 (25–52)	40 (28–55)	47 (28–60)	48.5 (25–52)	47 (28–60)
Profession, n (%)							
Registered nurse	67 (67)	16 (70)	1 (10)	14 (93)	14 (78)	16 (73)	6 (50)
Nurse assistant	12 (12)	1 (4)	2 (20)	0 (0)	1 (6)	2 (9)	6 (50)
Physician	20 (20)	6 (26)	6 (60)	1 (7)	3 (17)	4 (18)	0 (0)
Other profession*	1 (1)	0 (0)	1 (10)	0 (0)	0 (0)	0 (0)	0 (0)
Work experience, years, n (%)							
≤10	32 (32)	14 (61)	1 (10)	7 (47)	6 (33)	1 (5)	3 (25)
11–20	35 (35)	6 (26)	6 (60)	4 (27)	5 (28)	10 (45)	4 (33)
>20	33 (33)	3 (13)	3 (30)	4 (27)	7 (39)	11 (50)	5 (42)

Values are n (%) unless otherwise stated.

* One physiotherapist answered the survey and was included in the analysis.

**Figure 1:**
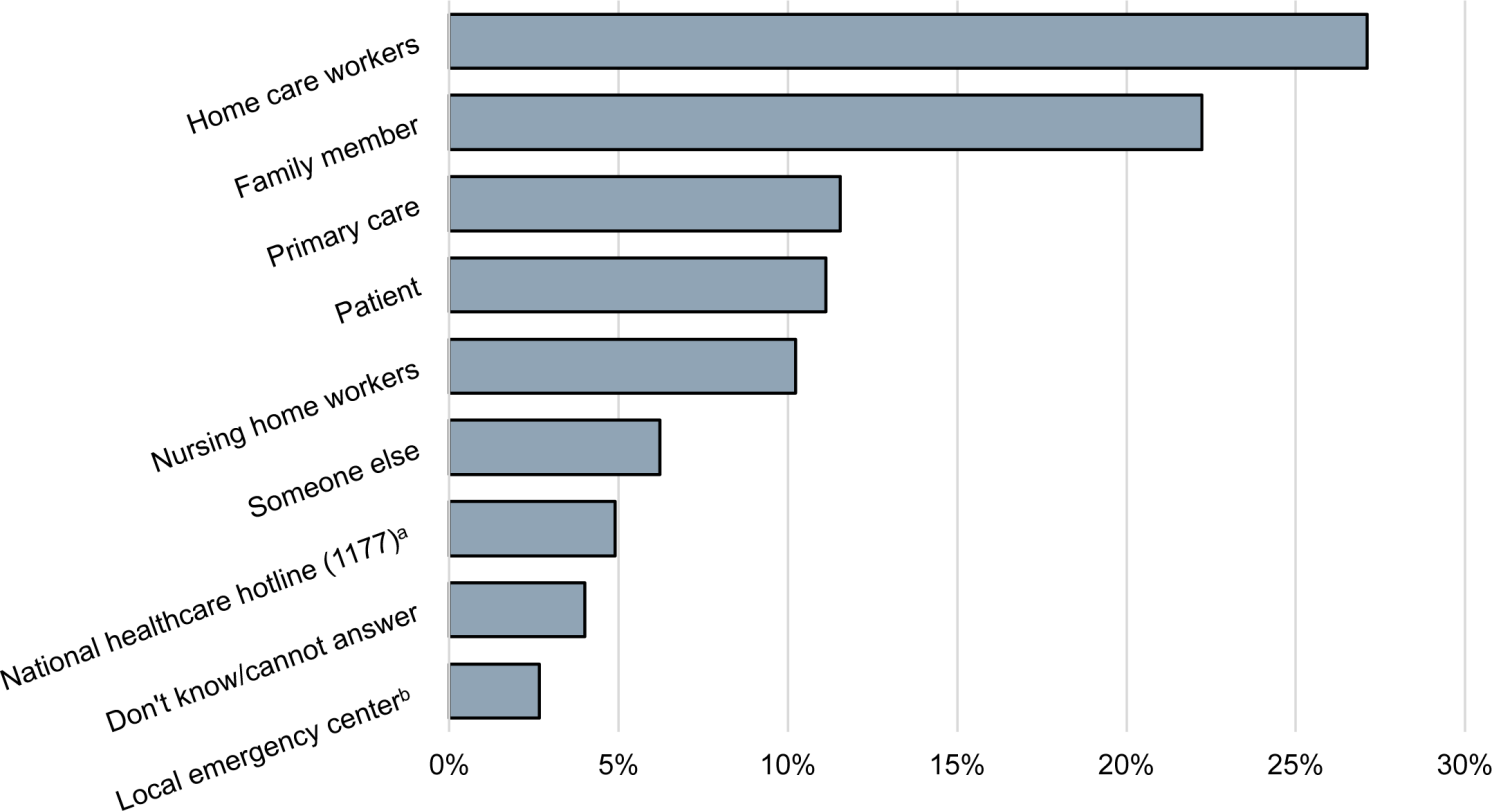
Who refers older adults with multiple long-term conditions to the emergency department? Respondents’ perspectives. Survey question: Who is most likely to initiate an emergency department visit for an older person with multiple long-term conditions? Percentages of all responses, multiple answers possible. n=100.*National telephone hotline available 24/7 for medical advice. †Local emergency clinics with limited diagnostic equipment.

All providers rated potentially avoidable ED visits as a significant problem, with a mean score of 6.6 on a 10-point Likert scale, and with ambulance services rating the highest (mean 8.2). Respondents perceived that referral to ED is most often initiated by home care workers (27%) followed by family members (22%) (figure 1). Collaboration between caregivers varied, rated poor between PC and ED and higher between PC and geriatrics or HHC out-of-hours (online supplemental material 3).

### Qualitative analysis of open-ended responses

**Figure 2:**
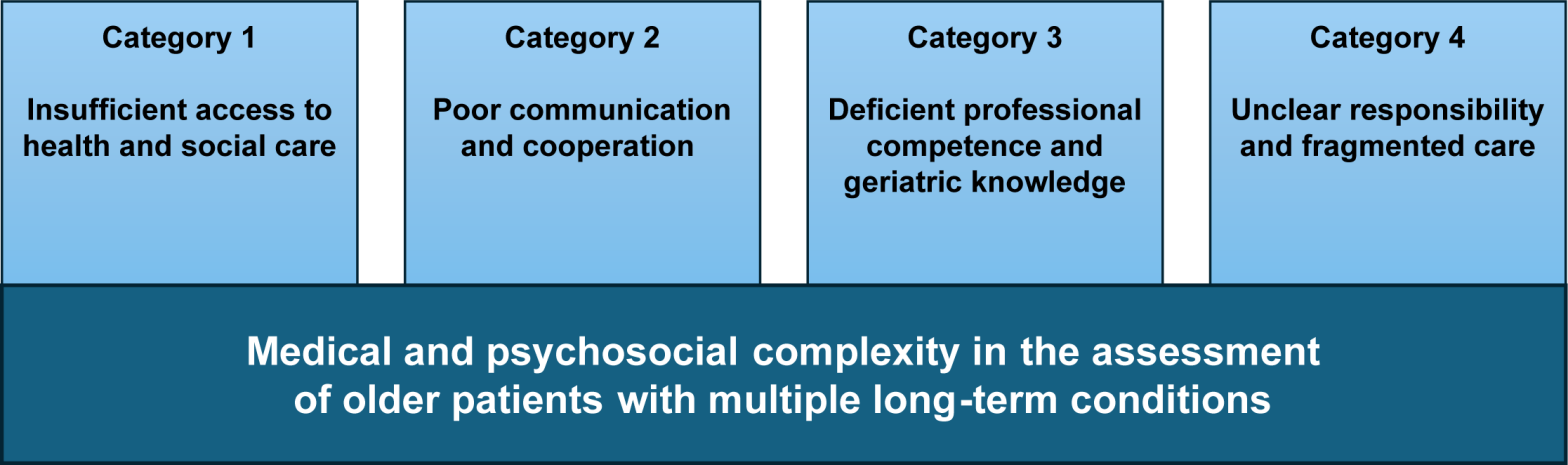
Qualitative theme and four categories of factors contributing to avoidable emergency department visits in older adults with multiple long-term conditions.

The analysis generated one theme—medical and psychosocial complexity complicate the assessment of older patients with multiple long-term conditions—and four categories of factors contributing to potentially avoidable ED visits: (1) insufficient access to health and social care, (2) poor communication and cooperation, (3) deficient professional competence and geriatric knowledge and (4) unclear responsibility and fragmented care (figure 2).

#### Overarching theme: medical and psychosocial complexity complicate the assessment of older patients with multiple long-term conditions

Healthcare professionals face complex challenges in the care of older adults. We interpreted this complexity as an underlying context influencing all factors, not as a target for intervention. It represents an inherent challenge when assessing older patients, particularly in acute situations.

All providers described that older adults with multiple long-term conditions have complex needs, symptoms that are difficult to interpret and communication challenges. Distinguishing acute symptoms from a patient’s habitual state was considered particularly difficult, for example, determining whether breathing difficulties were caused by heart failure, chronic obstructive pulmonary disease, infection or a combination. Frail patients often have abnormal vital signs—such as fluctuating blood pressure—further complicating assessment. Respondents also reported challenges in assessing patients with cognitive impairment who cannot clearly describe symptoms or medical history, leading to diagnostic and therapeutic delays. In addition, polypharmacy complicated evaluation, as symptoms may result from side effects. Finally, respondents from PC, advanced home-based healthcare, and basic home healthcare described ethically challenging situations when patients with cognitive decline refuse help, sometimes leading to acute deteriorations.

Older, frail patients often have nonspecific symptoms even if they have an underlying acute disease. For example, a subdural hematoma may present solely with slight personality changes or increased confusion. As mentioned, an accident with a fall can be a sign of something else. (ED)

They are vulnerable because they aren’t always lucid, and they can’t speak for themselves, they often don’t want to bother others with their symptoms. (PC)

#### Category 1: insufficient access to health and social care

Insufficient access to healthcare, municipal home care and nursing homes and the limited availability of home assessments contribute to ED visits.

##### Primary care

Lack of access to PC was commonly reported across all providers, with long waiting times for PC appointments and patients, families, home care workers and other healthcare professionals having difficulties reaching PC when needed. Long waiting times may cause ED visits, especially if patients view their condition as urgent, or if it worsens during the delay.

Healthcare in the patient’s home. Respondents described an urgent need for increased access to healthcare in the patient’s home—both on a regular basis and in emergency situations. They expressed that HHC may allow early detection of health deteriorations and timely treatment, prevent the exacerbation of chronic conditions and reduce the need for emergency care. Further, a nurse or physician assessment in the patient’s home in case of acute deterioration was described as a valuable alternative to ED visits. However, home visits were limited or often unavailable. Respondents from ambulance services, advanced home-based healthcare and HHC reported difficulties arranging physician assessments at home, especially after hours, often necessitating ED transfers.

Home care and nursing homes. Respondents reported that inadequate municipal home care, due to limited time with patients, causes unmet needs, increased anxiety and ED visits. Access to nursing homes was limited, forcing frail patients to remain at home even when home care no longer can provide sufficient support, leading to ED visits when the situation escalated.

Medical equipment. Ambulance services and PC staff described that lack of appropriate medical equipment led to ED referrals, such as complications with urinary catheters that could not be managed at home. Similarly, professionals from geriatric wards described that admitted patients needed to be transferred to the ED due to limited access to investigations such as CT scans and materials such as urinary catheters.

Hospital care. Respondents described that direct transfers from home to geriatric wards might reduce the frequency of ED visits, but this was often hampered by shortage of geriatric hospital beds. Lack of hospital beds also caused premature discharges from hospital care which in turn led to repeated ED visits.

They never get the time they need. Not because the will isn’t there, but because the staff does not have enough time. In the PC practice they barely have time to enter the physician’s office before their 10 minutes are up. If they spill their breakfast in bed, the home care workers don’t have time to change the sheets. If they need longer time in the bathroom the home care workers become stressed, as, they may only have a few minutes time for a check-up at that moment. It’s terrible to see. (advanced home-based healthcare)

(…) since we do not have the opportunity to perform, for example, urgent laboratory tests or ECGs in HHC, it can sometimes feel unnecessary to send a patient to the ED, when you know they often have to wait a long time at the ED. (PC)

### Category 2: poor communication and cooperation

Deficiencies in communication, cooperation and information transfer contribute to potentially avoidable ED visits.

#### Between professionals

Poor communication and collaboration were commonly mentioned. Ambulance professionals stressed the importance of direct contact with PC, while ED professionals called for better communication and case discussions, noting referrals for non-acute issues such as a lingering cough. Conversely, respondents from PC described difficulties reaching hospital-based specialists to discuss complex cases. Cases were described where a patient’s wish to decline treatment was not communicated to relevant healthcare providers, leading to ED referrals out-of-hours. Communication was further complicated by separate management of municipal home care and regional healthcare services, leaving home care workers unaware when patients have 24/7 advanced home-based healthcare.

#### Between professionals, patients and families

Respondents described that family decisions may lead to potentially avoidable ED visits when relatives request hospital evaluation despite the patient’s wish to stay home or contrary to professional advice against transfer. Moreover, patients and families may have been given ambiguous information, felt ignored or misunderstood, which in turn led to feelings of insecurity resulting in an ED visit.

#### Using and sharing medical records

Respondents found the medical record to be difficult to access, with limited functionality and poor usability. Ambulance services lack access to shared medical records, potentially risking patient safety. Furthermore, the use of multiple information systems and excessive documentation hinder effective use, complicating coordination.

It’s unknown to the ambulance staff what the latest care has been like, are we dealing with a new condition, or something chronic? Has the disease already been assessed and deemed harmless? We are groping in the dark. (ambulance services)

Too much text in the medical records when we’ve switched to standardized terms and everybody fills in something, even if the information is useless. For example, “Fall risk assessment: performed”, without writing what was concluded. (PC)

Difficulties communicating with other stakeholders. A lot of responsibility is placed on the individual and their relatives. If this fails, it leads to chaos. (PC)

### Category 3: deficient professional competence and geriatric knowledge

Respondents described that care professionals in general may lack knowledge about the complexity of older adults’ care.

#### Knowledge about care of frail older adults

Respondents emphasised that PC, HHC, municipal home care, nursing homes and even geriatric clinics may lack sufficient geriatric knowledge, leading to ED referrals for vague long-term symptoms or routine procedures such as catheter changes. Home care workers may call an ambulance even when healthcare at home would suffice, possibly reflecting gaps in competence. Conversely, delayed treatment was described to result in serious outcomes, such as infections that progress to sepsis. Geriatric ward professionals described that ambulance services may underestimate the severity of patients’ conditions resulting in incorrect triage. ED and ambulance respondents also expressed that nursing home and home care workers may lack training to assess emergencies, increasing the risk of misjudgements. Language barriers among home care workers were described as further hindering effective cooperation. Perceptions of insufficient competence were reported across all healthcare settings but were most prominently expressed by respondents working in emergency and hospital services, including ambulance services, ED, advanced home-based healthcare and geriatrics. These respondents described situations where patients were referred with conditions perceived as manageable at other levels of care, or where initial assessments were considered inadequate.

#### The right level of competence in the right place

Respondents described that staff with adequate competence may not be accessible. For example, a nursing home resident with erysipelas was unnecessarily sent to the ED after a registered nurse suspected deep vein thrombosis—a referral considered avoidable by the respondent had a physician been on-site. Respondents also emphasised that increased formal geriatric competence is needed among PC staff as well as in home care and nursing homes. Employing nurse assistants with formal nursing education in nursing homes and home care may contribute to better care.

This applies to patients in nursing homes – where doctors and nurses associated to nursing homes often make an inadequate or no assessment of the patient before they send the patient to the ED (for example with a lower urinary tract infection with normal vital parameters). This only leads to increased confusion, risk for healthcare-associated infections and it often ends with the patient getting a “ticket” back to the nursing home after a brief assessment (but often long waiting hours in the ED). (ED)

Lack of knowledge about the needs and conditions of the geriatric patient, polypharmacy, age discrimination. Reduced quality of life due to insufficient resources or inappropriate resources. Despite the frailty and complexity of the ageing person the symptoms of diseases are not prioritized or misinterpreted in older adults. (ambulance services)

### Category 4: unclear responsibility and fragmented care

Respondents from all providers stressed the need for a holistic approach instead of fragmented care. However, unclear responsibilities remain a key factor contributing to ED visits.

#### Follow-up and care transitions

Respondents from geriatrics, ambulance services, HHC and PC described that follow-up from PC after hospital care was unreliable and inconsistent. Examples include nurses who struggle to reach physicians, patients with chronic pain who do not receive adequate treatment and patients with long-term conditions such as heart failure who do not receive appropriate follow-up. Deficient follow-up may force patients to seek emergency care for symptoms that could have been managed in an outpatient setting.

#### Treatment plans

Respondents described a lack of distinct treatment plans acceptable to both patients and family members. For example, a nursing home resident with known anaemia was sent to the ED despite receiving prior transfusions at the nursing home; a treatment plan could have avoided the referral. Professionals from the ED, geriatrics and ambulance services expressed that palliative care often was initiated too late, resulting in excessive treatments and investigations that may increase suffering. They also reported that patients sometimes were excluded from decision-making or sent to the ED against their wishes.

#### During hospital admissions

Respondents described that patients were incompletely assessed during hospitalisations and discharged before recovery. Hospital treatment focused on the primary reason for admission, but older adults’ multiple conditions and symptoms were insufficiently addressed. For example, changes in long-term medications may not be initiated or monitored in hospital, leaving follow-up to PC. Such fragmented care and early discharge may result in readmissions and repeat ED visits.

Short hospital admissions lead to a lot of things needing to be done once the patient is discharged, if this is the case resources need to be available. For example, prompt follow-up by a PC physician. Many patients report being told they would be followed up by their PC physician upon discharge, but this does not happen. (geriatrics)

Patients are bounced between different departments = no one takes responsibility for the individual’s care. (HHC out-of-hours)

When the patient has seen different physicians over a certain period (one year). Some patients have been prescribed different medications by different physicians if no one has conducted a medication review. The patient ends up with medications they do not actually need. It becomes a vicious cycle and they don’t feel well, some of them end up taking Paracetamol for life. That’s one example. (HHC out-of-hours)

### Differences in provider perspectives

**Figure 3:**
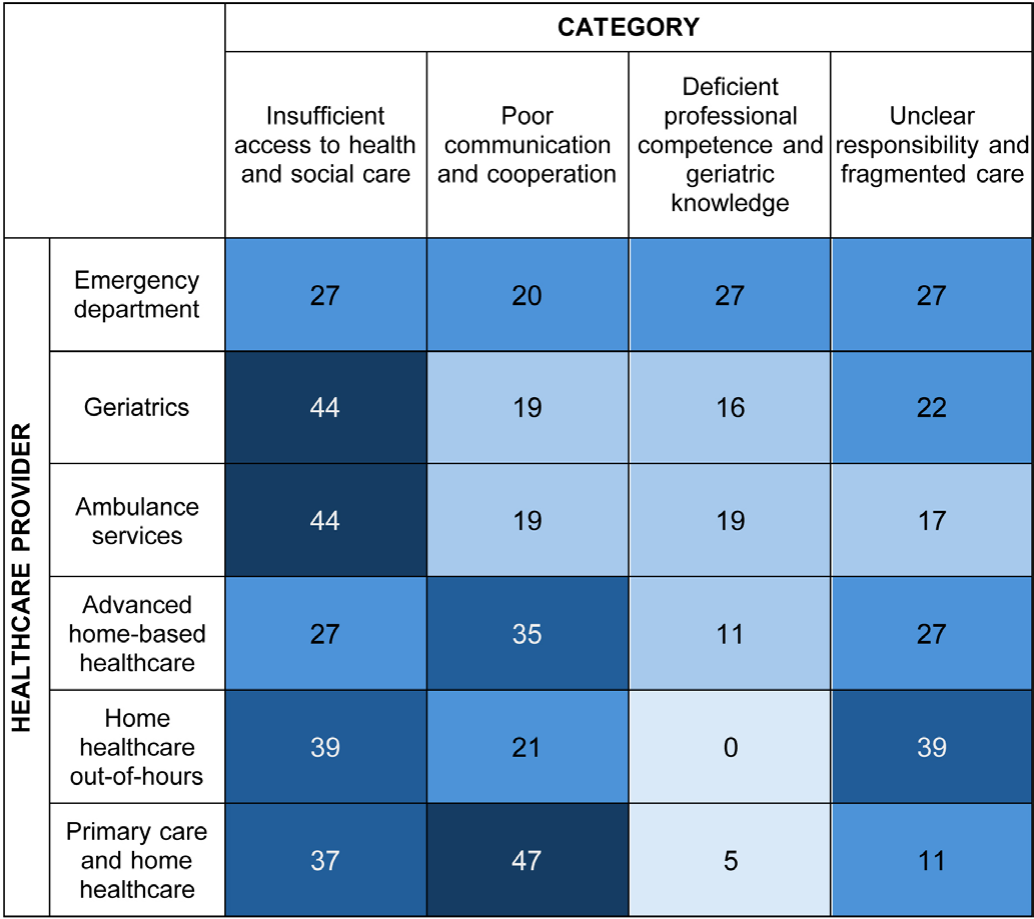
Differences in provider perspectives on reasons for emergency department visits, shown as proportions of meaning units* per category (columns) and care provider (rows) (darker shades indicate higher percentages). *Entire or part of open-ended response assigned to a category, one response may yield multiple meaning units, each respondent answered three questions.

Insufficient access to care was frequently mentioned by respondents from all providers and was the category most frequently mentioned by geriatrics and ambulance professionals (figure 3). HHC respondents working out-of-hours mentioned unclear responsibility more than other providers. PC responses focused mainly on communication, with few mentions of knowledge and responsibility.

### Patient case

To illustrate how multiple contributing factors may interact in a single potentially avoidable ED visit, we present a clinical case based on a real scenario described by a respondent.

A frail older man with dementia, living in a nursing home and receiving tube feeding via gastrostomy (PEG) was referred to the ED after the connector to the tube broke. On arrival, the ED staff discovered that the connector was not available at nighttime and that tube feeding was supportive rather than life-sustaining. The patient could return home, and nursing home workers contacted the hospital the following morning.

We interpret the causes of this ED visit as interconnected. Nursing home workers assessed the situation without realising that a non-functioning PEG was not life-threatening. This lack of awareness may reflect both insufficient competence and limited access to nurses and physicians. Additionally, staff did not consult the ED to discuss management. The decision to send the patient to the ED rather than attempt resolution at home may also indicate unclarities in responsibility for care.

## Discussion

### Main findings

This survey explored contributing factors to potentially avoidable ED visits in older adults with multiple long-term conditions as reported by healthcare professionals from six care providers across in- and outpatient care. All providers considered potentially avoidable ED visits to be a significant problem, with home care workers perceived as initiating most ED referrals. Qualitative analysis generated an overarching theme—medical and psychosocial complexities in the assessment of older patients with multiple long-term conditions—and four categories of factors contributing to potentially avoidable ED visits: (a) insufficient access to health and social care, (b) poor communication and cooperation, (c) deficient professional competence and geriatric knowledge and (d) unclear responsibility and fragmented care. While insufficient access was highlighted by all providers, PC respondents emphasised communication and collaboration more than the other categories. ED visits were commonly attributed to multiple factors that coexisted and interacted.

The identified categories also reflect an interplay between human and organisational factors which often co-occur and reinforce each other. Categories may reflect both dimensions. For example, unclear responsibility may be organisational in nature, when roles and structures are insufficiently defined, but may also reflect human factors, when individual healthcare professionals do not assume assigned responsibility. The data in this study do not allow us to fully disentangle these dimensions in all cases; however, this distinction is important to explore further in the design of system changes and healthcare interventions. Our findings align with previous research^18–20 31–33^ indicating that organisational deficiencies,^18 19 31–33^ poor collaboration and communication,^18 19 31–33^ limited access and continuity of outpatient care^18 19 31–33^ and unclear patient responsibility^20 33^ contribute to ED visits that might be avoided. Our findings also suggest potential differences in provider perspectives: ED professionals focused on improving access, while PC professionals emphasised collaboration and HHC out-of-hours unclear responsibility, possibly reflecting their different roles and experiences. Further investigating these differences may be important when designing interventions,^34^ as it highlights the need to consider variation in perspectives.

Several of the factors contributing to ED visits hold potential as components of an intervention. First, respondents emphasised the need to improve overall access to care, particularly PC, through timely home assessments. This is supported by previous research highlighting that access to PC is important to reduce ED visits.^35–37^ Ensuring access to continuous HHC to prevent exacerbations of long-term conditions, alongside emergency assessments by nurses and physicians to evaluate the need for ED referral, is essential. Reviews of home-based PC models in the USA^38^ and systematic reviews of hospital-avoidance interventions^14^ indicate that home visits can lower healthcare utilisation among older adults. Respondents also described a lack of basic medical equipment for simple procedures, such as urinary catheter management. Ensuring availability of such essential supplies is important for efficient care at home instead of transfer to the ED and may require attention at the organisational level.

Second, respondents—particularly from PC—highlighted the need for better collaboration between healthcare providers. ED-PC collaboration was rated as poor, and respondents from both expressed a desire for increased communication, which might reduce ED visits in cases of unclear urgency. Collaboration could also provide alternatives to ED visits, for example by enabling PC and geriatrics to arrange direct admissions to a geriatric ward when hospital admissions are needed but not urgent. Direct admissions have been associated with shorter hospital stays, fewer post-acute transfers and lower costs, though evidence is limited and selection bias may exist.^39 40^ Barriers include a lack of outpatient physicians to assess patients at home, hospital bed shortages and deficient collaboration between outpatient services and geriatrics. An alternative to direct admissions is advanced home-based healthcare, providing hospital-level care in the patient’s home, which is associated with lower mortality, reduced readmissions and higher levels of patient satisfaction compared with in-patient care.^41^

Formalised collaboration between healthcare providers could be a key component of a complex intervention aimed at reducing potentially avoidable ED visits, decreasing fragmentation of care and better aligning with person-centred care goals. However, improving collaboration remains a longstanding challenge, particularly in an urban setting such as Stockholm where many providers are involved in the care of older adults. Despite shared electronic health records, respondents in the current study reported persistent communication difficulties, partly due to information overload. Previous research indicates that effective collaboration depends not only on information-sharing systems but also on trust, shared understanding and clearly defined responsibilities across organisations.^42^ These findings suggest that interventions should go beyond technical solutions to also foster interprofessional relationships. Potential interventional components include structured opportunities for repeated interaction with assigned individuals to enhance the development of professional familiarity, support communication and joint decision-making in fragmented care systems.^42^ For example, this could involve direct consultation pathways between PC and geriatrics or ED for pre-referral case discussions.

Third, home care workers were perceived by respondents to most often initiate ED referrals. Respondents described cases where ambulances were called because HHC visits were unavailable or home care did not know whom to contact. This aligns with previous interviews with home care workers reporting that they recognise early signs of illness but lack support from PC, leading them to call ambulances even when HHC might have been sufficient.^43 44^ Home care workers often have the most comprehensive insight into the older adult’s condition when no relatives are present in the patient’s immediate environment or when the individual is unable to report on or fully understand their own health status.^43 44^ Their role in ED referrals highlights the need to integrate health and social care, as recommended by previous research and WHO policy.^45 46^

Fourth, respondents from ED, ambulance services, advanced home-based healthcare and geriatrics identified the lack of expertise and knowledge about geriatric patients in both healthcare, municipal home care and nursing homes as a cause of ED visits. However, while others’ lack of competence may be perceived as the primary cause when looking back, it is important to consider whether perceived insufficient competence may, in some cases, also be shaped by structural factors such as staffing shortages and resource availability. In contrast, respondents from PC and HHC less often emphasised competence, instead highlighting access and collaboration. This suggests that involving professional groups from both emergency and PC in intervention development may improve shared understanding of the problem.

Respondents highlighted that home care and nursing home workers may lack the knowledge and competence needed to care for older adults with complex care needs, which can lead to medical misjudgements. Home care workers do not always receive the proper training for the tasks they are meant to perform,^47 48^ and their education is commonly unregulated.^49^ They also face increasing demands at work when ageing-in-place policies result in a higher number of older adults with complex care needs living at home,^50^ and they may be delegated tasks that have previously been performed by healthcare.^51^ Notably, home care workers themselves express the need for further geriatric education, ideally delivered by the PC nurses they wish to collaborate with.^43 44^ Geriatric education for home care workers may be a key component of interventions to reduce ED visits in older adults, given its role in supporting sustained behaviour change.^52^

### Strengths and limitations

Including healthcare professionals from six in- and outpatient providers offers a comprehensive understanding of possible factors contributing to potentially avoidable ED visits in older adults with multiple long-term conditions. Although the inclusion criterion for professional experience was set relatively low at 6 months experience to facilitate recruitment, the majority of respondents were experienced healthcare workers; nearly 70% had over 10 years of work experience. Physicians, nurses and nurse assistants were represented. Perspectives from home care and nursing home workers, patients and family caregivers are not included in this study, but we have explored them in previous research, providing complementary insights.^43 44^

Surveys are effective for collecting large amounts of data, but they do not allow follow-up questions to explore responses in depth. Responses in this study varied from a few words to half an A4 page. Shorter responses are harder to interpret. However, open-ended questions allowed respondents to express their thoughts freely and highlight what they deemed most important, aligning with the study aim.

A methodological consideration is defining the target population as ‘older adults with multiple long-term conditions’ rather than ‘frail older adults’. In hindsight, a more precise use of ‘frail older adults’ could have improved conceptual clarity. However, the responses indicate that participants generally interpreted the term in line with the intended clinical group.

We aimed to include respondents across the care chain to get a comprehensive view via quota sampling, but quota sampling does not guarantee a representative sample,^23^ as selection relies on researcher judgement. Purposeful sampling^53^ via key contacts was initially attempted, but low response rates led us to distribute the survey link broadly. Although fewer than the planned 20 respondents were recruited from some providers, merging the results allowed the qualitative analysis to capture a wide range of perspectives. However, as some subgroups were as few as 10 providers, this limitation should be considered when interpreting the results. Consequently, comparisons across providers should be interpreted with caution.

This study investigates avoidable ED visits in urban and suburban settings; therefore, the transferability of the findings to rural settings may be limited. In urban settings, coordination among multiple healthcare providers is more likely to contribute to avoidable ED visits, whereas rural settings may be more strongly influenced by staffing shortages and limited healthcare resources. Recruitment through key contacts and managers may have influenced participant selection, potentially overrepresenting individuals with greater interest in the topic. This is an inherent challenge when recruiting for both surveys and qualitative research.^54^ At the same time, the recruitment strategy aimed to identify information-rich participants. Registered nurses comprised 67% respondents, but all targeted professional groups were represented. This distribution should be considered when interpreting transferability of the findings.

As some providers had as few as 10 respondents, the quantitative results should be interpreted as hypothesis-generating rather than generalisable. Counting meaning units can highlight differences in provider focus, but variations in response length may affect proportions, and results should be interpreted within the broader qualitative analysis.

## Conclusions

Caregivers from both in- and outpatient settings view potentially avoidable ED visits among older adults with multiple long-term conditions as a pressing concern arising from multiple interacting factors. Many of these factors are modifiable through interventions, such as expanding home-based care, strengthening collaboration between PC, ED, geriatric clinics and advanced home-based healthcare and providing targeted geriatric training for home care workers—who have the most frequent contact with many older adults and are seen as often initiating ED referrals. Intervention designers should consider differing provider perspectives to increase feasibility and acceptability.

## Supplementary material

10.1136/bmjopen-2026-121668 online supplemental file 1

10.1136/bmjopen-2026-121668 online supplemental file 2

10.1136/bmjopen-2026-121668 online supplemental file 3

## Data Availability

Data are available upon reasonable request.
