# Supplementary material for: Understanding potentially avoidable emergency department visits in older adults: a survey with healthcare professionals across in- and outpatient care in Sweden
Source: BMJ Open. 2026 Aug 14;16(8):e121668. doi: 10.1136/bmjopen-2026-121668 (PMC13479525; doi:10.1136/bmjopen-2026-121668)
Supplement: Supplementary file 1 [file bmjopen-16-8-s001.pdf]

Supplementary material 1 – Web Survey

Survey on preventing emergency department visits for older community-dwelling adults.

Cover letter

Would you like to participate in a research project that aims to prevent emergency department visits for older adults with complex health conditions?

About the Study

This survey aims to increase knowledge on how to prevent and reduce unnecessary emergency department visits for older adults with multiple long-term conditions. It also seeks to better understand the challenges faced by different healthcare professionals in caring for this patient group. Participation involves answering an online questionnaire, which takes approximately 15–20 minutes.

Your responses will be anonymized, and your personal data will be handled in accordance with the General Data Protection Regulation (GDPR). Only the responsible researchers, who are bound by confidentiality, will have access to the research material.

The principal investigator for this project is the Academic Primary Health Care Centre in the Region Stockholm. If you have any questions about the study, please contact xxx.

Click the link below to access the survey.

Thank you for your participation!

The survey consists of 11 questions where we aim to learn from your experiences.

In the open-ended questions, we encourage you to describe situations from your clinical practice, preferably with concrete examples (without mentioning any names).

"**Older adults with multiple long-term conditions"** refers to older individuals with complex medical and care needs.

To proceed with the survey, please provide your consent below.

I consent to my anonymized responses being collected and shared with researchers at Karolinska Institutet.

- o Yes
- o No (Survey will close)

1. I work at

- o Home health care/primary care
- o Primary care
- o Emergency department
- o Hospital-at-home services (ASIH)
- o Geriatrics
- o Home health care out-of-office hours
- o Another workplace, name which ___________

2. How significant is the problem of older adults with multiple long-term conditions visiting the emergency department when such visits could have been prevented?

Click on the line below and select the number that best reflects your assessment.

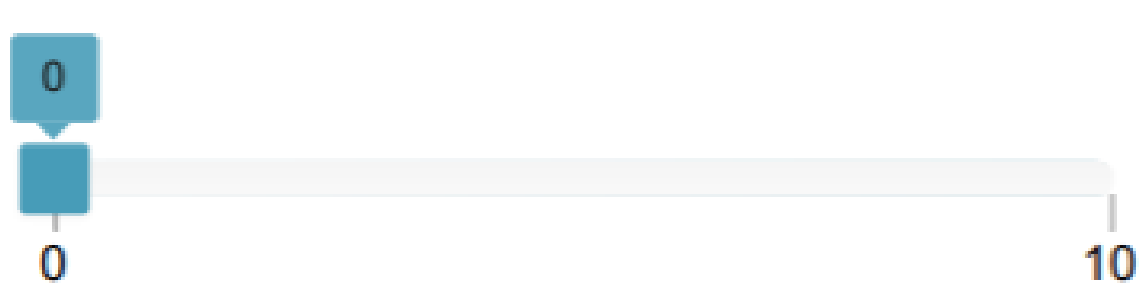

0 = Not a problem 10 = A significant problem

- o I don't know

3. Describe a patient case with an older adult with multiple long-term conditions where it would have been possible to prevent an ED visit. What should have been done differently, and how?

You can write as much as you like; the field expands automatically. We greatly appreciate detailed stories.

4. Who is most likely to initiate an ED visit for an older person with multiple long-term conditions?

Multiple answers possible.

- o The patient
- o Family member
- o Home care staff
- o Nursing home staff
- o Primary care
- o Local emergency center** (Närakut)
- o National healthcare hotline (1177)*
- o Someone else, who? ____________
- o Don't know/cannot answer

**National telephone hotline available 24/7 for medical advice.*

***Local emergency clinics with limited diagnostic equipment*

5. How does your collaboration with other care providers work regarding older adults with multiple long-term conditions?

Check the options that best apply.

"Collaboration" refers to actively exchanging information or planning care with other services or organizations regarding the older adult.

	No collaboration	Does not work at all	Works partly	Works well	Works very well
**Emergency department**	○	○	○	○	○
**Ambulance services**	○	○	○	○	○
**Hospital-at-home services**	○	○	○	○	○
**Geriatrics**	○	○	○	○	○
**Home care**	○	○	○	○	○
**Municipality/care administrator**	○	○	○	○	○
**Home health care out-of-office hours**	○	○	○	○	○
**Primary care rehabilitation/occupational therapist**	○	○	○	○	○
**Primary care rehabilitation/dietician**	○	○	○	○	○
**Primary care rehabilitation/physiotherapist**	○	○	○	○	○
**Nursing home**	○	○	○	○	○

6. What changes need to be made in healthcare to ensure that older adults with multiple long-term conditions avoid unnecessary ED visits?

You can write as much as you like; the field expands automatically

7. Describe three or more things that you find difficult or challenging in working with older adults with multiple long-term conditions.

You can write as much as you like; the field expands automatically

A few questions about you

8. I am

- Female
- Male
- Other
- Don't want to say

9. I am born in year

- Options 1937-2005
- Don't want to say

10. I am an educated

- Nurse assistant
- Registered Nurse
- Physician
- Other profession, which one? ___________

11. My work experience is

- Less than 1 year
- 1-5 years
- 6-10 years
- 11-15 years
- 16-20 years
- 21-25 years
- 26-30 years
- 31-35 years
- 36-40 years
- 41-45 years
- 46-50 years
- More than 50 years

Other comments

You can write as much as you like; the field expands automatically
