# Supplementary material for: Understanding potentially avoidable emergency department visits in older adults: a survey with healthcare professionals across in- and outpatient care in Sweden
Source: BMJ Open. 2026 Aug 14;16(8):e121668. doi: 10.1136/bmjopen-2026-121668 (PMC13479525; doi:10.1136/bmjopen-2026-121668)
Supplement: Supplementary file 2 [file bmjopen-16-8-s002.pdf]

Supplementary Material 2 – Example of qualitative analysis coding

Example of how open-ended survey responses were condensed, coded, and grouped into categories

**Meaning unit**	**Condensed meaning unit**	**Codes**	**Category**
Urinary catheter blockage requiring a visit to the ED. There should be better access to managing this issue in nursing homes and at home for patients with urinary catheters. (ambulance services)	Simpler conditions, such as catheter blockages, should be addressed at home or in nursing homes.	Catheter blockages managed at home/nursing homes	Insufficient access to healthcare, home care and nursing homes
No contact pathway to the responsible PC physician (ambulance services)	No contact pathway to PC physician	No contact pathway to PC	Poor communication and cooperation
The lack of a plan for the elderly patient's various potential needs. When conditions or symptoms change, it often leads to starting from scratch, leaving the patient feeling insecure. (HHC out-of-office hours)	The absence of a plan for various potential needs. When the condition changes, everything starts from scratch, leaving the patient feeling insecure.	Inadequate planning for potential deterioration.	Unclear responsibility and fragmented care

Three examples of the qualitative content analysis process, from meaning unit to category.
