# Supplementary material for: Understanding potentially avoidable emergency department visits in older adults: a survey with healthcare professionals across in- and outpatient care in Sweden
Source: BMJ Open. 2026 Aug 14;16(8):e121668. doi: 10.1136/bmjopen-2026-121668 (PMC13479525; doi:10.1136/bmjopen-2026-121668)
Supplement: Supplementary file 3 [file bmjopen-16-8-s003.pdf]

Online Resource 3 – Survey question 3: Collaboration

Question 3: How does your collaboration with other care providers work regarding older adults with multiple long-term conditions?

Darker shades indicate better collaboration. Left: responding care provider; Right: care providers rated for degree and quality of collaboration

On the left, the responding care provider; on the right, the care providers whose degree and quality of collaboration were rated. n = 100 of 100

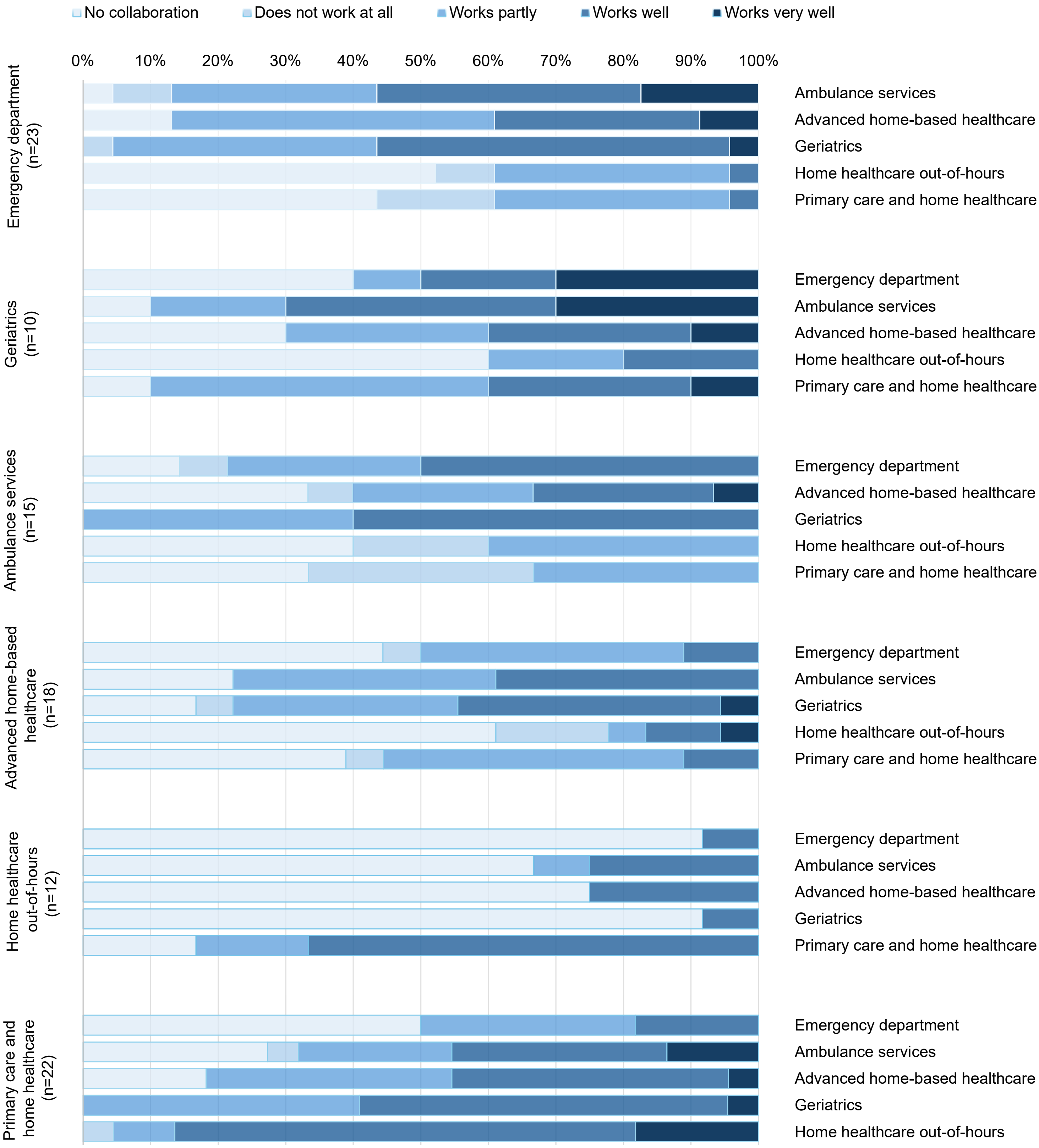
No collaboration
Does not work at all
Works partly
Works well
Works very well
0%
10%
20%
30%
40%
50%
60%
70%
80%
90%
100%
Emergency department (n=23)
Ambulance services
Advanced home-based healthcare
Geriatrics
Home healthcare out-of-hours
Primary care and home healthcare
Geriatrics (n=10)
Emergency department
Ambulance services
Advanced home-based healthcare
Home healthcare out-of-hours
Primary care and home healthcare
Ambulance services (n=15)
Emergency department
Advanced home-based healthcare
Geriatrics
Home healthcare out-of-hours
Primary care and home healthcare
Advanced home-based healthcare (n=18)
Emergency department
Ambulance services
Geriatrics
Home healthcare out-of-hours
Primary care and home healthcare
Home healthcare out-of-hours (n=12)
Emergency department
Ambulance services
Advanced home-based healthcare
Geriatrics
Primary care and home healthcare
Primary care and home healthcare (n=22)
Emergency department
Ambulance services
Advanced home-based healthcare
Geriatrics
Home healthcare out-of-hours
