# Supplementary material for: Understanding potentially avoidable emergency department visits in older adults: a survey with healthcare professionals across in- and outpatient care in Sweden
Source: BMJ Open. 2026 Aug 14;16(8):e121668. doi: 10.1136/bmjopen-2026-121668 (PMC13479525; doi:10.1136/bmjopen-2026-121668)
Supplement: Supplementary file 4 [file bmjopen-2026-121668.reviewer_comments.pdf]

PEER REVIEW HISTORY

BMJ Open publishes all reviews undertaken for accepted manuscripts. Reviewers are asked to complete a checklist review form and are provided with free text boxes to elaborate on their assessment. These free text comments are reproduced below.

ARTICLE DETAILS

Title (Provisional)

Understanding potentially avoidable emergency department visits in older adults: a survey with healthcare professionals across in- and outpatient care in Sweden

Authors

Bring, Hanna; Bergqvist, Monica; Gustafsson, Lars L; Veg, Anikó; Bohm, Katarina; Modig, Karin; Bastholm-Rahmner, Pia; Schmidt-Mende, Katharina

---

VERSION 1 - REVIEW

**Reviewer**	**1**
**Name**	**Källberg, Ann-Sofie**
**Affiliation**	**Dalarna University School of Education Health and Social Studies**
**Date**	**22-May-2026**
**COI**	**None**

This is a highly well-written study addressing a well-documented phenomenon, namely that older individuals are frequently admitted to emergency departments unnecessarily. The findings also provide a clear account of established factors that contribute to this occurrence. From this perspective, the study primarily serves to confirm existing knowledge. It would, however, strengthen the manuscript if the background or discussion sections elaborated on why this constitutes a problem. For instance, why should older individuals not be referred to emergency departments? Which types of visits should ideally be avoided?

I have several reflections regarding the methodology. In the development of the questionnaire, it is unclear why an educator, a behavioral scientist, and a pharmacist were included. Clarification of their specific contributions would be valuable. Furthermore, the inclusion criterion of at least six months of professional experience warrants further justification, as this may be considered a relatively limited period.

The description of a patient case also raises questions. Was the intention for this case to represent a fictitious or a real scenario? A more explicit rationale for this choice would enhance methodological transparency. Additionally, with regard to the professional groups

included in the study, it would be helpful to understand the rationale for including assistant nurses.

The results are presented in a clear and structured manner, and the identification of modifiable factors is a notable strength. Nevertheless, the discussion would benefit from more concrete examples, particularly regarding how communication between different healthcare providers could be improved. While previous efforts in this area have been made, often with limited success, it would be of interest to understand the authors' perspective on feasible strategies for improvement.

It is also somewhat unclear whether the aim of the study is to reduce emergency department visits among frail older adults specifically, or among older individuals more broadly. This raises the question of how "older individuals" are defined in the study, and whether such a definition was provided in the questionnaire.

Frail older adults, who are often characterized by multimorbidity, represent a particularly vulnerable group with a heightened risk of adverse events. It would therefore be valuable to clarify whether the broader objective of the study is to reduce such adverse outcomes in the longer term.

Overall, this is an interesting study that raises several important questions. Its contribution could be further strengthened by addressing and discussing some of the issues outlined above.

**Reviewer**	**2**
**Name**	**Stolker, Robert Jan**
**Affiliation**	**Erasmus MC, Anesthesiology**
**Date**	**02-Jun-2026**
**COI**	**None**

Thank you for asking me to review this paper.

The authors performed a survey across six in- and outpatient healthcare providers in Stockholm. The analysed the answers in order to find issues contributing to avoidable ED visits.

In my opinion is this topic key as avoidable ED visits harm patients and lead to substantial costs. My major concern is that I miss the context of the specific organisation of the Swedish healthcare, especially the relation between in and outpatient care.

The problem of avoidable ED visits is universal.

In my country (NL) triage during the day is done by GP's and during out off office hours bij triagists in GP-driven carepoints, who give advise what to do. More serious case are asked to

visit the GP while the more severe cases are referred to the ED. GP's and home care organisations play a pivotal role in out-of-hospital care.

A brief explanation will elucidate the bottlenecks caregivers face everyday, especially in frail patients with potential acute problems

Other concerns/suggestions

Methods

The study was done in urban and suburban areas of Stockholm. Are the described problems also an issue in rural areas and why?

What are "targeted healthcare professionals"? How are they selected? Is this a potential source of bias? "Respondents were recruited through key contacts..." and "Fewer than 20 respondents were included in four of six health care providers." Does this influence the answers?

What are the professional qualifications of primary health care workers providing basic health care during the day or out-of hours (HHC category 5 and 6)? What is the definition of home care workers? Are doctors involved?

Who pretested the survey? Are the results of the interview studies already available?

Can you specify who analysed the answers? On page 9 the authors mention that "Firts the free-text responses were read repeatedly (line 25). Is this done by the same person?

Who splited the responses containing multiple aspects. Was this done according to a predefined framework?

Results

Could the authors say something about the representativity of the respondents? This is at more importance as four out of six categories did not reach the goal of 20 respondents.

Category 1: I do not understand that patients have to be transferred for simple procedures with urinary catheters, EKG, blood taking simple ultrasound. Are these not available outside the hospitals or at an outpatient -basis? Or is this a result of legal rules?

Category 3:

Lack of geriatric and emergency skills (especially in frail patients) are not uncommon.

However, healthcare providers may emphasise these knowledge gaps in other health care providers. In this perspective the paragraph on differences in provider perspectives is short. Are more perspectives available?

In case of home care providers who call an ambulance fear for claims may perhaps be a reason for the reference to an ED Department.

Discussion

The discussion is organised according to the category framework.

Is it possible to distinguish between human factors (such as communication, education, etc) and more organisation issues?

Strenghts and limitation are well described.

VERSION 1 - AUTHOR RESPONSE

Reviewer: 1

Dr. Ann-Sofie Källberg, Dalarna University School of Education Health and Social Studies

Comments to the Author:

Comment 1. This is a highly well-written study addressing a well-documented phenomenon, namely that older individuals are frequently admitted to emergency departments unnecessarily. The findings also provide a clear account of established factors that contribute to this occurrence. From this perspective, the study primarily serves to confirm existing knowledge. It would, however, strengthen the manuscript if the background or discussion sections elaborated on why this constitutes a problem. For instance, why should older individuals not be referred to emergency departments? Which types of visits should ideally be avoided?

Authors' response:

Thank you for your positive and thoughtful comments, and for highlighting the need to better explain why further research on potentially avoidable ED visits among older adults is needed.

Older adults, particularly those with multiple long-term conditions and frailty, are vulnerable to adverse outcomes associated with ED visits and hospitalization. While ED care is essential in acute situations, potentially avoidable visits may expose patients to unnecessary risks, including functional decline, hospital-associated complications, fragmented care, and care that is not aligned with patients' preferences. At the same time, avoidable ED visits contribute to increased healthcare use and place additional strain on already burdened emergency services.

We have therefore expanded the Background section to provide a clearer rationale for why reducing potentially avoidable ED visits is important. We also highlight that, despite considerable research attention and multiple intervention studies, evidence regarding effective strategies remains inconclusive. The persistence of this problem underscores the need for further contextual and intervention-focused research. In this context, we believe that

our study contributes by exploring the perspectives of healthcare professionals across multiple healthcare providers involved in the care of older adults.

Comment 1, Background

Page 4, line 24 – page 5, line 1

While vital in emergencies, ED visits pose risks for frail older adults and are associated with complications such as infections, medication errors, functional decline and mortality[6–8]. The anticipated rise in ED visits due to an aging population[9,10] is a global public health concern, causing strain to hospitals and EDs[1–4,11,12].

Every fourth ED visit in older ~~patients~~ adults might be avoidable, either by care at another level or through more proactive outpatient care[12]. Since ED visits expose older adults to unnecessary risks[3,6,7], ~~but interventions aiming to~~ reducing them~~e such visits~~ has been a focus of previous research[13–15]. Yet, the interventions show inconsistent results[13–15].

Comment 2. I have several reflections regarding the methodology. In the development of the questionnaire, it is unclear why an educator, a behavioural scientist, and a pharmacist were included. Clarification of their specific contributions would be valuable.

Authors' response: Thank you for this important comment. We agree that it is valuable to clarify the role of the researchers in the development of the questionnaire.

The behavioural scientist (PB) has extensive experience in qualitative research and survey methodology and contributed to the design and refinement of the questionnaire. The pharmacist and the educator are senior researchers within our research group, both with experience in healthcare development and intervention work. They were involved in reviewing and pretesting the survey, with particular focus on ensuring clarity, relevance, and comprehensibility across professional groups.

Including team members from different disciplinary backgrounds was intentional, as it enabled us to incorporate perspectives beyond clinical practice alone. This helped to identify potential ambiguities and broaden the relevance of the questions. All members contributed through iterative discussions during the development and pretesting of the survey.

These clarifications have been added to the Methods section of the manuscript.

Comment 2, Methods

Page 6, line 11-15

The survey was developed by a panel of physicians, registered nurses, a pharmacist, a pedagogue, and a behavioural scientist. All panel members had expertise in healthcare development processes, except for HB (MD, PhD student). The panel contributed to development through iterative discussions on content, clarity and relevance from both clinical and methodological perspectives.

Comment 3. Furthermore, the inclusion criterion of at least six months of professional experience warrants further justification, as this may be considered a relatively limited period.

Authors' response: Thank you for this important reflection. We acknowledge that a minimum of six months of professional experience may be considered relatively short. However, this threshold was chosen pragmatically to ensure feasibility of recruitment while still including participants with sufficient exposure to the clinical context under study.

In practice, the majority of respondents reported considerably longer professional experience, with 70% reporting more than 10 years of professional experience. We consider this to have strengthened the relevance and depth of the data. We have added a clarification of this point in the Strengths and limitations section:

Comment 3, Strengths and limitations

Page 22, line 1-4

Although the inclusion criterion for professional experience was set relatively low at 6 months experience to facilitate recruitment, tThe majority of respondents were experienced healthcare workers;, nearly 70% had over 10 years of work experience.

4. The description of a patient case also raises questions. Was the intention for this case to represent a fictitious or a real scenario? A more explicit rationale for this choice would enhance methodological transparency.

Authors' response: Thank you for this important comment. The patient case was included to illustrate how multiple contributing factors can interact in a single potentially avoidable ED visit. The case is based on a real clinical scenario described by a respondent but has been modified to preserve anonymity and protect confidentiality.

We agree that the rationale for including the case should be made more explicit and have clarified this in the manuscript. The purpose of the case is to provide a concrete example of how different factors may interact and contribute to potentially avoidable ED visits among older adults with complex care needs.

Comment 4, Results

Page 18, line 11-14

To illustrate how multiple contributing factors may interact in a single potentially avoidable ED visit, we present a clinical case based on a real scenario described by a respondent. ~~Potentially avoidable ED visits commonly result from multiple interacting causes, as exemplified in the following patient case:~~

5. Additionally, with regard to the professional groups included in the study, it would be helpful to understand the rationale for including assistant nurses.

Authors' response: Thank you for this critical comment. Nurse assistants were included as they represent a key professional group in care of older adults, particularly in home healthcare. They work very closely with frail older patients and are often directly involved in situations that may lead to emergency department referrals. Additionally, their perspectives are unexplored compared to registered nurses and physicians.

In one of the included healthcare providers, home healthcare out-of-hours, nurse assistants constitute a main part of the care team, and 50% of respondents were nurse assistants. Their perspectives were therefore considered highly relevant for understanding factors contributing to potentially avoidable ED visits.

We have added a rationale for the professions we targeted with the survey in the Methods section:

Comment 5, Methods

Page 8, line 1-4

The main professional groups targeted were physicians, registered nurses and nurse assistants. They are all key healthcare providers involved in the care of older adults with multiple long-term conditions and in decisions on care related to ED visits.

Comment 6. The results are presented in a clear and structured manner, and the identification of modifiable factors is a notable strength.

Authors' response: Thank you for this positive comment and for highlighting the strengths of our study.

Comment 7. Nevertheless, the discussion would benefit from more concrete examples, particularly regarding how communication between different healthcare providers could be improved. While previous efforts in this area have been made, often with limited success, it would be of interest to understand the authors' perspective on feasible strategies for improvement.

Authors' response: We thank the reviewer for this pertinent suggestion. The issue is well known, and as noted, previous efforts have often had limited success. We agree that this remains a significant challenge. We have therefore elaborated in the Discussion on potential feasible components of an intervention, while also acknowledging the inherent complexity of improving cross-organisational communication and collaboration in this context.

Comment 7, Discussion,

Page 20, line 16-31

Formalized collaboration between ~~PC, ED, geriatrics and advanced home-based~~ healthcare providers could be a key component of complex interventions aimed at reducing potentially avoidable ED visits, decrease fragmentation of care and better align with person-centred care goals. However, improving collaboration remains a longstanding challenge, particularly in an urban setting such as Stockholm where many providers are involved in the care of older adults. Despite shared electronic health records, respondents in the current study reported persistent communication difficulties, partly due to information overload. Previous research shows that effective collaboration depends not only on information systems but also on trust, shared understanding, and clearly defined responsibilities between organisations. These

findings suggest that interventions should extend beyond technical solutions to also foster interprofessional relationships. Potential interventional components include structured opportunities for repeated interaction with assigned individuals to enhance the development of professional familiarity, support communication and joint decision-making in fragmented care systems. For example, this could involve direct consultation pathways between primary care and geriatrics or ED for pre-referral case discussions.

Comment 8. It is also somewhat unclear whether the aim of the study is to reduce emergency department visits among frail older adults specifically, or among older individuals more broadly. This raises the question of how "older individuals" are defined in the study, and whether such a definition was provided in the questionnaire.

Authors' response: Thank you for the comment. We agree that the aim and target population required further clarification.

When the survey was developed in 2022, we used the Swedish term *multisjuka äldre*, translated in this study as "older adults with multiple long-term conditions." We acknowledge that this term is not fully equivalent to "frail older adults" in a strict clinical definition, as multimorbidity alone does not necessarily imply frailty. However, in the clinical context in which the survey was conducted, the term is closely associated with frailty and complex care needs.

To ensure a shared understanding among respondents, we provided a definition on the survey cover page. We agree that this could have been formulated more precisely, and in hindsight, explicitly using "frail older adults" may have improved conceptual clarity. Nevertheless, based on the content of the responses, participants appear to have interpreted the target group in line with the intended clinical population.

We have clarified this both in the Methods section and in the Strengths and Limitations section of the manuscript, as indicated below.

Comment 8a, Methods

Page 6, line 20 - page 7, line 2

The cover page provided a definition of the target population to ensure common understanding:

"Older adults with multiple long-term conditions" refers to older individuals with complex medical and care needs.

Comment 8b, Strengths and Limitations

Page 22, line 13-17

A methodological consideration is defining the target population as "older adults with multiple long-term conditions" rather than "frail older adults. In hindsight, a more precise use of "frail older adults" could have improved conceptual clarity. However, the responses indicate that participants generally interpreted the term in line with the intended clinical group.

9. Frail older adults, who are often characterized by multimorbidity, represent a particularly vulnerable group with a heightened risk of adverse events. It would therefore be valuable to clarify whether the broader objective of the study is to reduce such adverse outcomes in the longer term.

Authors' response: Thank you for this comment. This is our aim of this study and if this is not clear it is important to clarify. We have added a short section to the end of the background to further clarify that this is the long-term goal of the study.

Comment 9, Background

Page 5, line 13-15

Overall, more interventional research targeting frail older adults is crucial to prepare healthcare systems for an ageing population[15] in general, and to reduce the adverse outcomes associated with ED visits in particular.

10. Overall, this is an interesting study that raises several important questions. Its contribution could be further strengthened by addressing and discussing some of the issues outlined above.

Authors' response: Thank you for this helpful and constructive overall assessment of our study. We appreciate the reviewer's insightful comments, which have helped us improve the clarity and methodological reporting of the manuscript. We have carefully addressed all

specific points raised and revised the manuscript accordingly to strengthen its contribution further.

Reviewer: 2

Dr. Robert Jan Stolker, Erasmus MC

Comments to the Author:

Comment 1. Thank you for asking me to review this paper.
The authors performed a survey across six in- and outpatient healthcare providers in Stockholm. The analysed the answers in order to find issues contributing to avoidable ED visits.
In my opinion is this topic key as avoidable ED visits harm patients and lead to substantial costs.

Authors' response: Thank you for this positive assessment of our study and for highlighting the relevance of the topic.

Comment 2. My major concern is that I miss the context of the specific organisation of the Swedish healthcare, especially the relation between in and outpatient care.
The problem of avoidable ED visits is universal.
In my country (NL) triage during the day is done by GP's and during out off office hours bij triagists in GP-driven carepoints, who give advise what to do. More serious case are asked to visit the GP while the more severe cases are referred to the ED. GP's and home care organisations play a pivotal role in out-of-hospital care.
A brief explanation will elucidate the bottlenecks caregivers face everyday, especially in frail patients with potential acute problems

Authors' response: Thank you for this important comment. We agree that clearer contextual information regarding the organisation of the Swedish healthcare system, particularly the relationship between in- and outpatient care, is essential for interpreting the findings. In the Swedish system, the GP does not function as a gatekeeper to emergency care, which influences pathways to ED use greatly. We have therefore added a Box explaining contextual background to improve clarity.

Comment 2, Methods

Page 5, line 29

The study was conducted in urban and suburban areas of Stockholm (population 2.5 million). In Sweden, health and social care is tax funded. Regions organise healthcare, while municipalities organise social care. Primary care does not function as a gatekeeper for emergency department (ED) visits, and patients can access EDs without referral. Municipalities employ home care workers who provide support with instrumental and personal activities of daily living. They may be formally trained nurse assistants or untrained personnel.

The survey included healthcare professionals from six key healthcare providers:

1. **Emergency department (ED):** 24/7 emergency care with full diagnostic capacity; no referral required.
2. **Geriatrics:** Hospital-based geriatric wards for acute care, with limited access to advanced diagnostics, particularly out of hours.
3. **Ambulance services:** Community emergency assessments 24/7, mainly staffed by registered nurses.
4. **Advanced home-based healthcare:** 24/7 services providing hospital-level care at home, including intravenous therapy and blood transfusions, with on-call physicians.
5. **Primary care (PC):** Clinic-based care and basic home healthcare delivered by teams of GPs, registered nurses, and nurse assistants. Home healthcare includes wound care, medication administration, and medical assessment and follow-up for homebound patients.
6. **Home healthcare out of hours (HHC):** Evening, night, and weekend services providing basic nursing care (e.g., wound care, medication administration), staffed by registered nurses and nurse assistants without on-call physicians.

Other concerns/suggestions

Methods

Comment 3. The study was done in urban and suburban areas of Stockholm. Are the described problems also an issue in rural areas and why?

Authors' response: Thank you for this question. The issue of potentially avoidable ED visits among older adults with multiple long-term conditions is not specific to urban settings. However, the underlying organisational conditions may differ between urban, suburban, and rural healthcare contexts.

In rural areas, challenges related to staffing and recruitment of healthcare professionals may have a greater impact on accessibility. At the same time, coordination and communication across providers may in some cases be less complex due to fewer healthcare organisations involved. In contrast, there are over 200 primary care practices in Stockholm region, and patients are not always registered with a practice geographically close to them. Together with multiple hospital sites, this contributes to fragmented care pathways and a situation in which GPs may have less continuity of responsibility compared to rural settings.

As our study was conducted in urban and suburban settings, we are not able to make direct comparisons with rural healthcare systems. We have therefore chosen not to expand this discussion in depth in the manuscript. However, we acknowledge that contextual differences are likely important and should be considered when interpreting findings and when planning complex healthcare interventions.

Comment 4. What are "targeted healthcare professionals"? How are they selected? Is this a potential source of bias? "Respondents were recruited through key contacts..." and "Fewer than 20 respondents were included in four of six health care providers." Does this influence the answers?

Authors' response: Thank you for this critical comment. We agree that the recruitment process required further clarification.

The main professional groups targeted were physicians, registered nurses, and assistant nurses, as these professions are closely involved in the care of older adults and decision-making related to emergency department visits.

As this study primarily aimed to generate qualitative insights, the intention was not to obtain a representative sample but rather to recruit respondents with sufficient experience and willingness to elaborate on the topic. Nevertheless, this recruitment strategy may have affect transferability of findings. For example, healthcare professionals with a particular interest in the topic or stronger opinions regarding avoidable ED visits may have been more likely to be invited or choose to participate. It is also possible that managers and key contacts influenced which individuals were approached.

Regarding the inclusion of fewer than 20 respondents in four of the six healthcare providers, we acknowledge that this may have affected the range of perspectives captured. However, respondents described a broad variety of experiences and deficiencies across the healthcare system. In line with the concept of information power, we assessed the data as sufficiently rich and diverse to address the study aim.

To improve transparency, we have expanded both the Methods section and the discussion of strengths and limitations to describe the recruitment process and its potential implications for transferability. We already discuss the effects of the small sample size in the strengths and limitations section.

Comment 4a, Methods

Page 8, line 1-33

The inclusion criterion of respondents was at least six months of working experience. Respondents were recruited through managers and key contacts within each provider., The managers were recruited via authors' using professional networks. Key contacts were informed about the study and asked to invite 20 eligible healthcare professionals with experience of the issue and interest in elaborating their responses to complete the survey. Staff were invited to participate via e-mail. No incentive was offered. The survey was initially sent to individuals chosen by the key contact; if less than 20 respondents were recruited, it was subsequently spread broadly via key contacts.

The survey was initially sent to the respondents between September 2022 and January 2023. A cover letter with information about the study, its confidentiality, the voluntary nature of participation and informed consent was placed included at the beginning of the survey (Additional File 1). Although 20 respondents per target group initially agreed to participate, response rates varied. After five reminder e-mails to eligible respondents, additional respondents were recruited through the same key contacts, who distributed the survey link directly.

Initially, PC professionals were excluded, as their perspectives were to be studied separately, but following preliminary analysis they were included to gain a more comprehensive understanding. As PC practices are typically smaller organizations, a different recruitment strategy was used. The survey link was disseminated widely distributed to PC professionals via key contacts and authors' professional networks between November 2023 - February 2024.

Due to recruitment difficulties and time restrictions, fewer than 20 respondents were included in four of six healthcare providers. Because the survey link was widely disseminated, the total number of eligible recipients could not be established, and a response rate could therefore not be calculated. Using the concept of information power[24], we assessed the data as sufficiently rich in information in relation to the study's aim, providing adequate nuance and diversity for analysis.

Comment 4b, Strengths and limitations

Page 22, line 21-29

Although fewer than the planned 20 respondents were recruited from some providers, merging the results allowed the qualitative analysis to capture a wide range of perspectives. However, as some subgroups were as few as 10 providers, this limitation should be considered when interpreting the results. Consequently, comparisons across providers should be interpreted with caution.

Recruitment through key contacts and managers may have influenced participant selection, potentially overrepresenting individuals with greater interest in the topic; however, this is an inherent challenge when recruiting for both surveys and qualitative research[53]. At the same time, the recruitment strategy aimed to identify information-rich participants.

Comment 5. What are the professional qualifications of primary health care workers providing basic health care during the day or out-of hours (HHC category 5 and 6)? What is the definition of home care workers? Are doctors involved?

Authors' response: Thank you for this important request for clarification. We have revised the Methods section to more clearly specify the professional composition of the included healthcare services, the definition of municipal home care workers, and the role (or absence) of physicians within each setting. We have added a box which explains the settings, and clarifies that primary care home healthcare involves physicians, registered nurses, and assistant nurses, while HHC out-of-hours is staffed only by registered nurses and assistant nurses with no physicians involved. See answer Comment 2.

6. Who pretested the survey?

Authors' response: The study was pretested by three healthcare professionals, one from ED, one from geriatrics and one from advanced home-based healthcare. These individuals were selected to represent different clinical contexts to ensure clarity and face validity of the questionnaire. This is stated in the survey development section.

Comment 6, Methods

Page 6, line 15-17:

To enhance face validity, the survey was pretested on three professionals (from ED, geriatrics, and advanced home-based healthcare), resulting in minor adjustments of style and grammar.

7. Are the results of the interview studies already available?

Authors' response: One study is published: Mannheimer S, Bergqvist M, Bastholm-Rahmner P, Gustafsson LL, Vég A, Schmidt-Mende K. Experiences of patients receiving Home Care and living with polypharmacy: a qualitative interview study. BJGP Open. 2022 Jun;6(2):BJGPO.2021.0181. doi:10.3399/BJGPO.2021.0181 PubMed PMID: 35135816; PubMed Central PMCID: PMC9447318. We have added a reference.

The other interview study referred to in page 9, line 2-3 is currently undergoing revision and will hopefully soon be published in BMJ open.

Comment 8. Can you specify who analysed the answers? On page 9 the authors mention that "Firts the free-text responses were read repeatedly (line 25). Is this done by the same person?

Authors' response: Thank you for this clarification request. All answers were read repeatedly by PBR, MB and HB, all three researchers collaborated on the analysis. A clarification has been added to the methods section:

Comment 8, Methods

Page 9, line 17-22

Primary qualitative analysis was conducted by PBR (senior researcher with expertise in qualitative research, behavioural scientist) and MB (senior researcher with expertise in qualitative research, registered nurse). After recruitment of primary care respondents HB (PhD student, MD, resident in general practice) took main responsibility for splitting responses into meaning units, coding and analysis, with regular meetings and support from MB and PBR.

9. Who splited the responses containing multiple aspects.

Authors' response: The splitting of responses containing multiple aspects was primarily carried out by HB (PhD student, MD, resident in general practice), who had main responsibility for the final coding and analysis after recruitment of primary care respondents. This process was undertaken in close collaboration with MB and PBR, who had conducted the initial phase of analysis prior to primary care recruitment. All decisions regarding splitting

of meaning units, coding, and interpretation were continuously discussed in regular analytic meetings to ensure analytical rigour.

This clarification has been added to the Methods section of the manuscript, see response Comment 8.

10. Was this done according to a predefined framework?

Authors' response: No predefined framework was used. The analysis followed an inductive approach to analysis, codes, and categories were derived from the data without imposing a predetermined coding frame or theory.

This is mentioned in the methods section. We have added a methodological reference to on the inductive approach for clarity.

Comment 10, Methods

Page 9, line 13-14

The open-ended questions were analysed using qualitative content analysis[25], with an inductive approach[26].

Results

11. Could the authors say something about the representativity of the respondents? This is at more importance as four out of six categories did not reach the goal of 20 respondents.

Authors' response: Thank you for this important comment. We agree that the issue of representativeness requires clarification. The strength of this study is that is includes 6 different healthcare providers all providing important perspectives on reasons for ED visits.

This study includes both quantitative and qualitative analyses. Due to the limited number of respondents within each healthcare provider, the quantitative findings should not be interpreted as generalisable, as also stated in the Strengths and Limitations section.

The qualitative findings should likewise not be interpreted as representative of all healthcare professionals within the included providers. We acknowledge that fewer than 20 respondents were included in four of the six healthcare providers, which may have influenced the breadth of perspectives captured. However, respondents described a wide range of experiences and

system-level deficiencies across settings. In line with the concept of information power, we assessed the dataset as sufficiently rich and diverse in relation to the study aim. Nevertheless, any comparisons across groups should be interpreted with caution.

Representativeness is an inherent challenge in all survey studies, as respondents who choose to participate are likely to be more motivated or interested in the topic, which can influence both the quantitative generalisability and qualitative transferability of the results.

We have clarified these issues further in the Strengths and Limitations section of the manuscript:

Comment 11, Strengths and limitations

Page 22, line 21-31

Although fewer than the planned 20 respondents were recruited from some providers, merging the results allowed the qualitative analysis to capture a wide range of perspectives. However, as some subgroups were as few as 10 providers, this limitation should be considered when interpreting the results. Consequently, comparisons across providers should be interpreted with caution.

Recruitment through key contacts and managers may have influenced participant selection, potentially overrepresenting individuals with greater interest in the topic; however, this is an inherent challenge when recruiting for both surveys and qualitative research[53]. At the same time, the recruitment strategy aimed to identify information-rich participants. Registered nurses comprised 67% respondents, but all targeted professional groups were represented. This distribution should be considered when interpreting transferability of the finding.

12. Category 1: I do not understand that patients have to be transferred for simple procedures with urinary catheters, EKG, blood taking simple ultrasound. Are these not available outside the hospitals or at an outpatient -basis? Or is this a result of legal rules?

Authors' response: This is a matter of how the Swedish healthcare is organized. For instances, in this survey there were descriptions of transfers to ED for simple procedures from geriatric hospitals, ambulance services and nursing homes. Both professionals from geriatrics and ambulance service expressed their frustration that these simple materials were not available at their location, geriatric clinic and in the patients home respectively. This is

not a result of legal rules, but a definite area for improvement in healthcare provision in the Stockholm Region.

A few lines have been added to the discussion clarifying this.

Comment 12, Discussion

Page 19, line 33 - page 20, line 3

Respondents also described a lack of basic medical equipment for simple procedures, such as urinary catheter management. Ensuring availability of such essential supplies is important for efficient care at home instead of transfer to the ED and may require attention at the organisational level.

13. Category 3:
Lack of geriatric and emergency skills (especially in frail patients) are not uncommon. However, healthcare providers may emphasise these knowledge gaps in other health care providers. In this perspective the paragraph on differences in provider perspectives is short. Are more perspectives available?

Authors' response: Thank you for this interesting comment. We agree that differences in perspectives across provider groups are important to clarify. In our material, descriptions related to lack of geriatric and emergency competence were most frequently expressed by respondents working in emergency care and hospital settings, including ambulance services, ED, and geriatric inpatient services.

These perceptions should be interpreted in relation to each group's position in the care chain, and may reflect differences in exposure, responsibility, and organisational context. It is therefore important to consider whether what is perceived as insufficient competence may, in some cases, also be influenced by structural factors such as staffing levels and resource availability.

We have clarified this in the Results section, and reflected further on the importance of perspectives on competence in the discussion section.

Comment 13a, Results

Page 16, line 3-8

Perceptions of insufficient competence were reported across all healthcare settings but were most prominently expressed by respondents working in emergency and hospital services, including ambulance services, ED, advanced home-based healthcare and geriatrics. These respondents described situations where patients were referred with conditions perceived as manageable at other levels of care, or where initial assessments were considered inadequate.

Comment 13b, Discussion

Page 21, line 9-18

Fourth, respondents from ED, ambulance services, advanced home-based healthcare and geriatrics identified the lack of expertise and knowledge about geriatric patients in both healthcare, municipal home care and nursing homes as a cause of ED visits. However, while others' lack of competence may be perceived as the primary cause when looking back, it is important to consider whether perceived insufficient competence may, in some cases, also be shaped by structural factors such as staffing shortages and resource availability. In contrast, respondents from PC and HHC less often emphasized competence, instead highlighting access and collaboration. This suggests that involving professional groups from both emergency and PC in intervention development may improve shared understanding of the problem.

Comment 14. In case of home care providers who call an ambulance fear for claims may perhaps be a reason for the reference to an ED Department.

Authors' response: Thank you for this interesting observation. We think that home care workers need both adequate knowledge and appropriate support, and they should not be left responsible of complex medical decisions.

We find this result of our study particularly important, and our research group is currently engaged in a co-design intervention project aimed at strengthening collaboration between home care workers and primary care practices. The goal is to provide more timely medical support and thereby reduce situations in which home care workers are left without adequate guidance in clinical decision-making.

Discussion

Comment 15. The discussion is organised according to the category framework.

Is it possible to distinguish between human factors (such as communication, education, etc) and more organisation issues?

Authors' response: Thank you for this insightful and helpful comment. We agree that it is interesting to consider whether the identified categories can be distinguished as reflecting human factors versus organisational factors.

We have therefore added a reflecting paragraph in the discussion.

Comment 15, Discussion

Page 19, line 10-16

The identified categories also reflect an interplay between human and organisational factors which often co-occur and reinforce each other. Categories may reflect both dimensions. For example, unclear responsibility may be organisational in nature, when roles and structures are insufficiently defined, but may also reflect human factors, when individual healthcare professionals do not assume assigned responsibility. The data in this study do not allow us to fully disentangle these dimensions in all cases; however, this distinction is important to explore further in the design of system changes and healthcare interventions.

Comment 15. Strenghts and limitation are well described.

Authors' response: Thank you for this positive comment.

VERSION 2 - REVIEW

**Reviewer**	**1**
**Name**	**Källberg, Ann-Sofie**
**Affiliation**	**Dalarna University School of Education Health and Social Studies**
**Date**	**09-Jul-2026**
**COI**	

I am satisfied with the revisions you have made in response to my suggestions.

**Reviewer**	**2**

Name **Stolker, Robert Jan**

Affiliation **Erasmus MC, Anesthesiology**

Date **20-Jul-2026**

COI

The concerns/suggestions of my co-reviewer and me have been addressed properly with one exception. My comment #3 is addressed in the rebuttal letter but not in the text. It should be mentioned that this survey is done in a (suburban) setting and that a rural setting is not investigated. This is a limitation and perhaps it should even be mentioned in the title.

VERSION 2 - AUTHOR RESPONSE

Reviewer: 1

Dr. Ann-Sofie Källberg, Dalarna University School of Education Health and Social Studies
Comments to the Author:
I am satisfied with the revisions you have made in response to my suggestions.

Authors' response: Thank you. We are pleased that the revisions addressed your comments.

Reviewer: 2

Dr. Robert Jan Stolker, Erasmus MC
Comments to the Author:
The concerns/suggestions of my co-reviewer and me have been addressed properly with one exception. My comment #3 is addressed in the rebuttal letter but not in the text. It should be mentioned that this survey is done in a (suburban) setting and that a rural setting is not investigated. This is a limitation and perhaps it should even be mentioned in the title.

Authors' response: We are pleased that the reviewer considers that most of the comments have been adequately addressed. We also appreciate the remaining comment regarding the study setting and agree that the suburban setting of our study affects the transferability of the findings to rural contexts. To address this, we have included a paragraph in the Strengths and Limitation section elaborating on this.

We appreciate the suggestion to reflect the study setting in the title. After careful consideration, we revised the title to specify that the study was conducted in Sweden. However, we chose not to include "suburban" in the title to avoid making it unnecessarily

long, as the study setting is described in detail in the manuscript and its implications are now discussed in the *Strengths and Limitations* section.

page 23, line 9-13:

This study investigates avoidable ED visits in urban and suburban settings; therefore, the transferability of the findings to rural settings may be limited. In urban settings, coordination among multiple healthcare providers is more likely to contribute to avoidable ED visits, whereas rural settings may be more strongly influenced by staffing shortages and limited healthcare resources.
